# *Astragalus* Polysaccharides Inhibits Tumorigenesis and Lipid Metabolism Through miR-138-5p/SIRT1/SREBP1 Pathway in Prostate Cancer

**DOI:** 10.3389/fphar.2020.00598

**Published:** 2020-05-05

**Authors:** Shanqi Guo, Baojie Ma, Xingkang Jiang, Xiaojiang Li, Yingjie Jia

**Affiliations:** ^1^Department of Oncology, First Teaching Hospital of Tianjin University of Traditional Chinese Medicine, Tianjin, China; ^2^Department of Urology, The Second Hospital of Tianjin Medical University, Tianjin, China

**Keywords:** *Astragalus* polysaccharides, miRNA-138-5p, prostate cancer, SIRT1, SREBP1

## Abstract

*Astragalus* polysaccharides (APS) is a traditional Chinese medicine and have been proved to involve in multiple biological processes, including inflammation, metabolism, and carcinogenics. However, the specific mechanisms by which APS on prostate cancer (PCa) remains largely unknown. In the current study, we found APS greatly inhibited the proliferation and invasion of PCa cells in a dose-dependent and time-dependent manner *in vitro* and *in vivo*. In addition, cellular triglyceride and cholesterol levels were also decreased significantly under APS treatment. Microarray data revealed the SIRT1 expression was markably suppressed under APS exposure. Mechanistic studies demonstrated that over-expression of SIRT1 inhibits the expression and nuclear translocation of SREBP1 *via* activating AMPK phosphorylation to suppress lipid metabolism. Otherwise, knockdown of SIRT1 significantly promotes AMPK/SREBP1 signaling and its associated target genes. Besides, we also found miR-138-5p was greatly inhibited the SIRT1 expression to regulating cell metabolism by targeting its 3′UTR region. To summarize, our findings suggested that APS inhibits tumorigenesis and lipid metabolism through miR-138-5p/SIRT1/SREBP1 pathways in PCa.

## Introduction

Prostate cancer (PCa) is one of the most common malignancies in men worldwide. In the wake of the aging of population, the change of lifestyle and the development of new diagnosis methods and treatment technologies, the incidence of PCa is increasing yearly worldwide (Bashir, 2015; Hassanipour-Azgomi et al., 2016). Recent researches showed that the “Western diet” pattern with high cholesterol level was involved in the occurrence and progression of PCa. Therefore, reduce cholesterol level is of great significance for the prognosis of PCa patients (Schnoeller et al., 2017; Stopsack et al., 2017). Statins are currently used to prevent cardiovascular events and control cholesterol level. Unfortunately, statins have certain risks, such as liver damage and skeletal muscle damage (Liu et al., 2019a). Therefore, choosing a drug that can reduce cholesterol level as well as avoid adverse reactions to the greatest extent will have important and far-reaching significance for the treatment and prognosis of patients with PCa.

*Astragalus* is one of the largest genuses of flowering plants in the Leguminosae family. *Astragalus* genus is well known for their pharmacological properties, particularly hepatoprotective, immunostimulant, and antiviral activities. Modern medical studies indicated that *Astragalus* had anti-tumor, immune regulation, antioxidant stress and other pharmacological effects, and has been widely used in clinical medicine and biological fields (Li et al., 2014). *Astragalus* polysaccharides (APS), as one of the main effective components of *Astragalus*, have been reported to regulate the processes of inflammation, metabolism, and carcinogenes. Recent studies demonstrated that the application of APS restores the cytokine balance and inhibit the immune suppressive effects of Treg cells in the tumor micro-environment (Li et al., 2012). Besides, Li and colleagues also found that APS induced breast cancer cells apoptosis by activating macrophages to release Nitric Oxide (NO) and Tumor Necrosis Factor-α (TNFα) (Li et al., 2019). Furthermore, APS treatment also enhanced the chemo-sensitivity of hepatoma cell lines by inhibiting MDR1 and P-glycoprotein efflux pump function (Tian et al., 2012a). Chemo-sensitizing effect of APS was also found by modulating expression of Bax/Bcl-2 ratio, caspases and JNK pathway (Tian et al., 2012b; Zhou et al., 2017; Li et al., 2018). Recently, Liu et al. performed the network pharmacology with targetable screening from the Cancer Genome Atlas and identified the anti-proliferative effect of APS by regulating CCNB1, CDC6, and P53 in breast cancer (Liu et al., 2019b). Although the anti-tumor role of APS in multiple kinds of cancers has been studied, the molecular mechanism of APS in PCa has not been well recognized. Other than this, the possible role of APS in cancer lipid metabolism is still limited.

In the present study, we concluded that APS inhibits cell proliferation, invasion, and lipid metabolism *in vitro* and *in vivo*. Mechanistic studies demonstrated APS inhibited the progression of PCa and lipid metabolism by targeting miR-138-5p/SIRT1/SREBP1 pathways. Therefore, this study pointed out a new approach of APS to treat PCa from the perspective of lipid metabolism.

## Materials and Methods

### Cell Culture and Chemicals

Human PCa cell lines (PC3 and DU145) were purchased from the American Type Culture Collection. Cells were cultured in RPMI-1640 medium (Gibco) with 10% fetal bovine serum (Hyclone) and 100 U/ml penicillin-streptomycin (Hyclone). These cell lines were maintained at 37°C and 5% CO_2_ in a humidified incubator. APS (UV≥90.0%) was purchased from Solarbio Co. Beijing, China. The APS was dissolved in PBS to 10 mg/ml and then diluted with RPMI-1640 culture medium containing 10% Fetal Bovine Serum (FBS) at different concentrations and stored at 4°C for further experiments.

### Cell Growth, Colony Formation, and Invasion Assays

Cells were treated with different concentrations of APS (0, 1, 5, 10, 20, 40 mg/ml) and at different times (0, 24, 48, 72, 96 h). Cell proliferation was determined using the CCK-8 assay following the protocol of the manufacturer (Dojindo, Japan). Cells were cultured in six-well plates and incubated for 2 weeks, and then the colony formation was counted manually. Moreover, cell invasion was performed with Transwells assay coated with Matrigel.

### Microarray Analysis

PC3 cells were treated with APS at 10 mg/ml and negative control (NC) for 48 h. Then, total RNAs were extracted by using Trizol reagent (Invitrogen). The mRNA array data were analyzed by using the GeneSpring software V13.0 (Agilent). To select the differentially expression genes, we used threshold values of ≥2 and ≤2-fold change and a t-test *p* value of 0.05. Gene expression data are available in NCBI GEO database (No. GSE137486).

### Cell Transfection

Human SIRT1 cDNA were cloned into the pcDNA3.1 vector (Clonetech, USA). The miR-138-5p mimic and inhibitor and their NC were purchased from GenePharma (China). Plasmids and miRNA transfection were performed using Lipofectamine™ 2000 (Invitrogen, USA). SIRT1 shRNA constructs were cloned into lentiviral vector pLKO.1. Lentiviral particles were harvested and transduced in presence of polybrene (Sigma-Aldrich).

### Isolation of Total RNA and qRT-PCR

Total RNAs were isolated from harvested cells using Trizol reagent (Invitrogen). Two microgram RNA was reversed transcribed using the reverse transcriptase kit (TaKaRa). Quantitative PCR (qPCR) was conducted using the power SYBR Green master mix (Roche). GAPDH and U6 were respectively used as an internal control for mRNA and miRNA. The sequences were listed in **Supplemental Table 1**.

### Western Blot

Cells were harvested and lysed in RIPA buffer containing protease inhibitor (Solarbio). Total protein concentration was measured by the BCA assay kit (Solarbio). The protein labels were visualized using the ECL detection system (Thermo). The primary antibodies of SIRT1 (1:500), SREBP1(1:500), ACC (1:500), FASN (1:500), and GAPDH (1:1,000) were purchased from Proteintech.

### Luciferase Assay

Luciferase reporter activity was detected by using the dual-luciferase reporter assay system kit (Promega), according to the manufacturer’s protocol as described previously (Jiang et al., 2016).

### Measurements of Cellular Triglyceride and Cholesterol Levels

Cells were lysed and extracted according to procedures specified by individual commercial kits (Jiancheng, Nanjing). The cholesterol quantification kit and the triglyceride (TG) quantification kit were respectively used to quantify levels of cellular TGs and cholesterol levels.

### Tumor Xenograft Treatment Model

We selected male nude mice aged 6-weeks to construct *in vivo* model, and then randomly divided into three groups: NC group, low dose APS (LD-APS) group of 50 mg/kg, and high dose APS (HD-APS) group of 100 mg/kg. After 2 weeks of tumorigenesis, mice were given by gavage once per day, a total of 14 d. The mice were sacrificed 2 h after the last gavage, and measured their tumor growth. The animal protocol was also approved by the ethics committee of First Teaching Hospital of Tianjin University of Traditional Chinese Medicine.

### Statistical Analysis

Data was shown as the means ± standard error using SPSS 17.0 software. All experiments were independently performed in triplicate. Statistical analysis was performed by two-tailed Student’s t-test or one-way ANOVA test, and a *P*-value of <0.05 determined statistical significance.

## Results

### APS Inhibited Cell Proliferation and Lipid Metabolism in PCa Cells

**Figure 1:**
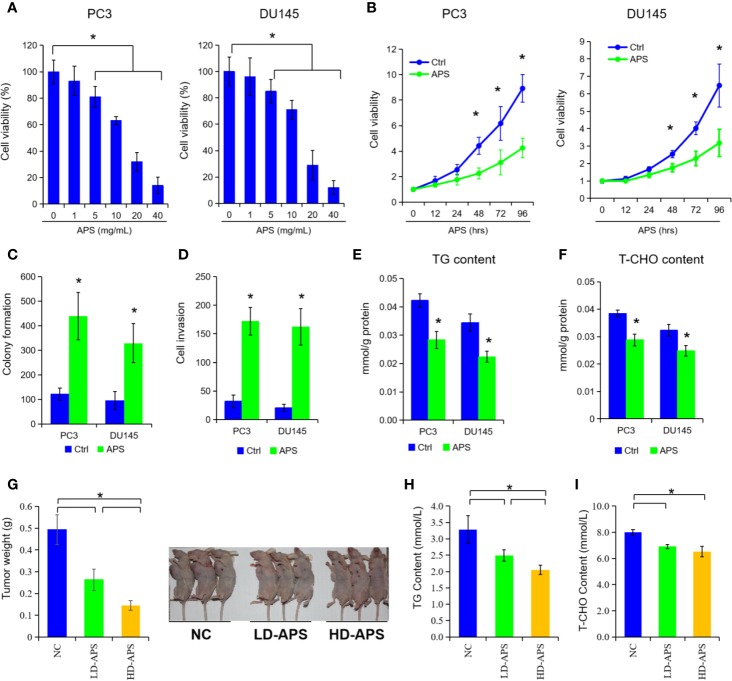
*Astragalus* polysaccharides (APS) inhibited cell proliferation and lipid metabolism in PCa cells. Cell viability were assessed by CCK-8 assay in PC3 and DU145 cells at different dose of APS **(A)**. Cell viability were examined in PC3 cells and DU145 cells under APS treatment at different time period **(B)**. Cell colony formation and cell invasion ability were evaluated in PC3 and DU145 cells under a dose of 5 mg/ml APS treatment **(C**, **D)**. The cellular triglyceride (TG) and total cholesterol (T-CHO) content were determined in PC3 and DU145 cells under a dose of 5 mg/ml APS treatment at 48 h **(E**, **F)**. The mean weight of the xenograft tumors, serum TG, and T-CHO content were detected in negative-control group (NC), low-dose APS group (LD-APS), and high-dose APS group (HD-APS) **(G**–**I)**. Asterisk (*) indicates P < 0.05.

To explore the anti-tumor function of APS in PCa cells, we selected two common cell lines (PC3 and DU145 cells) treated by APS at different doses and times. As depicted in **Figure 1A**, the growth ability of PC3 and DU145 cells was significantly suppressed under APS treatment at 48 h in a dose-dependent manner (P < 0.05). Then, we also found that APS exhibited a time-dependent cytotoxity in PC3 and DU145 cells at 5 mg/ml dosage (**Figure 1B**, P < 0.05). In addition, cell colony and cell invasion were evaluated with a dose of 5 mg/ml APS treatment at 48 h, respectively. The results showed that APS greatly inhibited the ability of cell colony and cell invasion by almost 40%, compared with the corresponding control cells (**Figures 1C, D**, P < 0.05). To our surprise, the cellular TG and cholesterol levels were also decreased in PC3 and DU145 cells under APS treatment (**Figures 1E, F**, P < 0.05). As for *in vivo* experiments, we established an animal model of PC3 treated with different concentrations of APS (i.e. NC, LD-APS, and HD-APS group). We found the tumor growth were inhibited by approximately 40% under APS treatment, and the serum TG and cholesterol were also suppressed in APS treatment (**Figures 1G–I**, P < 0.05). These data together demonstrated that APS decreases cell growth and lipid metabolism in PCa cells.

### APS Suppressed SIRT1 Expression to Inhibit Cellular Lipid Metabolism

**Figure 2:**
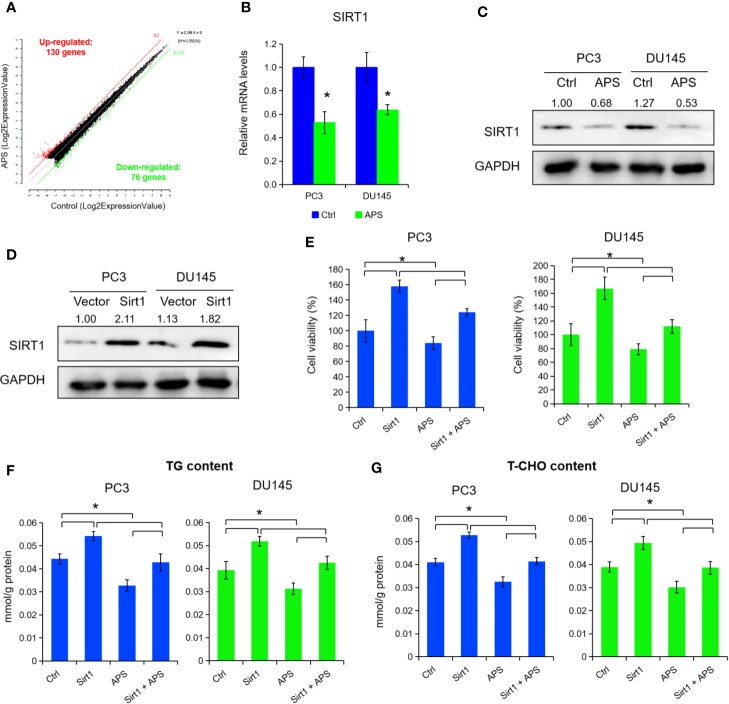
*Astragalus* polysaccharides (APS) suppressed SIRT1 expression to inhibit cellular lipid metabolism. The scatter plot showed 130 genes were up-regulated and 76 genes were down-regulated in PC3 cells under APS treatment (fold change ≥ ± 2) **(A)**. The mRNA and protein levels of SIRT1 were detected under APS treatment in PC3 and DU145 cells by qRT-PCR and Western blot assay **(B**, **C)**. The protein levels of SIRT1 were examined by Western blot assay in PC3 and DU145 cells under SIRT1 overexpression **(D)**. Cell viability, TG, and T-CHO were assessed in SIRT1 transfected PC3 and DU145 cells under APS treatment **(E–G)**. Asterisk (*) indicates P < 0.05.

To elucidate the underlying molecular role of APS in lipid metabolism, we performed a microarray analysis to detect different mRNA levels in PC3 cells. As shown in **Figure 2A** and **Supplemental Table 2**, the microarray data indicated that 206 genes were changed with a dose of 5 mg/ml APS treatment at 48 h, accordingly (130 up-regulation and 76 down-regulation, fold change ≥ ± 2). After critical literature review, SIRT1 was identified among them for further elevation. Consequently, we detected the mRNA and protein levels of SIRT1 expression under APS treatment, and found a dose of 5 mg/ml APS exposure significantly inhibited the SIRT1 levels in PC3 and DU145 cells (**Figures 2B, C**, P < 0.05). To further investigate its biological role in PCa, we transfected the SIRT1 and their NC plasmids in PCa cells. **Figure 2D** showed that the protein levels were increased more than 2.1 fold and 1.8 fold in SIRT1-transfected PC3 and DU145 cells. Cell proliferation assay indicated that over-expression of SIRT1 decreased the cell susceptibility to APS-induced cytotoxicity compared to those of control group (**Figure 2E**, P < 0.05). Besides, the cellular TG and cholesterol levels were also restrained in SIRT1-transfected PC3 and DU145 cells upon APS treatment (**Figures 2F, G**, P < 0.05). Together, the above data indicated a critical role of SIRT1 against APS-induced cytotoxity.

### SIRT1 Promotes the Expression of SREBP1 *via* Activating AMPK

**Figure 3:**
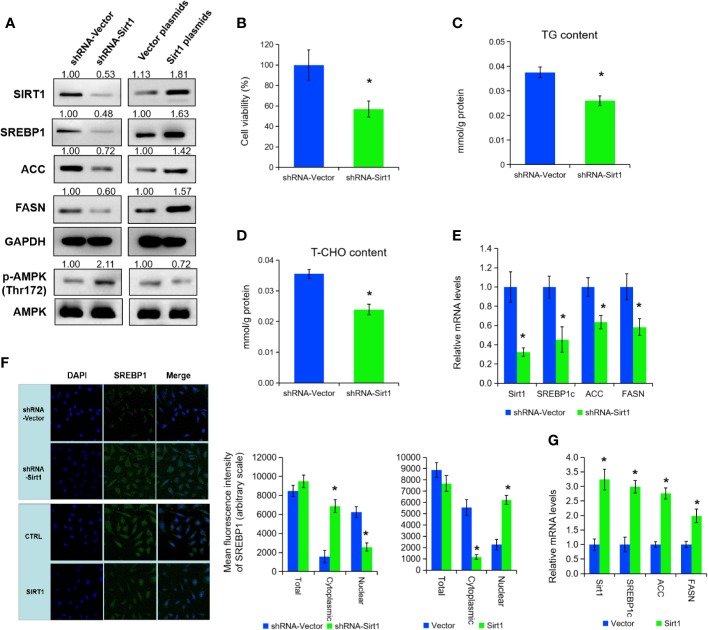
SIRT1 promotes the expression of SREBP1 *via* activating AMPK. The protein levels of SIRT1, SREBP1, ACC, FASN, p-AMPK, and AMPK were detected by Western blot assay in PC3 cells treated with shRNA-SIRT1 or SIRT1 plasmids **(A)**. Cell viability, TG, and T-CHO were assessed in SIRT1-knockdown PC3 cells **(B**–**D)**. The mRNA levels of SIRT1, SREBP1, ACC, and FASN were detected in PC3 cells after SIRT1 knockdown **(E)**. Confocal images (left panel) and quantitative fluorescence intensity (right panel) for SREBP1 (green) in PC3 cells transfected with shRNA-SIRT1 or SIRT1 plasmids, and DAPI (blue) stains nuclei **(F)**. The mRNA levels of SIRT1, SREBP1, ACC, and FASN were detected in PC3 cells upon SIRT1 overexpression **(G)**. Asterisk (*) indicates P < 0.05.

To further explore the mechanisms of SIRT1 in lipid metabolism under APS treatment, we inhibited SIRT1 expression by transfection of shRNA in PC3 cells (**Figure 3A**). Cell growth, cellular TG, and cholesterol level were prominently decreased under SIRT1 knockdown (**Figures 3B–D**, P < 0.05). Then, qRT-PCR and Western blot assay certified that the expression of SREBP1 and the associate target genes (e.g. ACC, FASN) were markedly decreased under the suppression of SIRT1 (**Figures 3A, E**, P < 0.05). Consistent with previous studies, the phosphorylation level of AMPK (Thr172) was elevated under SIRT1 knockdown, compared to those of control group (**Figure 3A**). Additionally, silencing of SIRT1 greatly increased the cytoplasmic/nuclear ratio of SREBP1 in PC3 cells (**Figure 3F**, P < 0.05). Besides, over-expression of SIRT1 decreased the AMPK phosphorylation levels, and thus inhibited the levels of SREBP1 and its cholesterol-related target gene expression (**Figures 3A, G**, P < 0.05). The nuclear translocation of SREBP1 was increased in SIRT1-transfected PC3 cells under APS treatment (**Figure 3F**, P < 0.05). Thus, we concluded that SIRT1 regulate the expression of SRBEP1 *via* activating AMPK to mediate the lipid metabolism of PCa cells.

### miR-138-5p Can Specifically Bind to SIRT1 to Promote Tumor Progression

**Figure 4:**
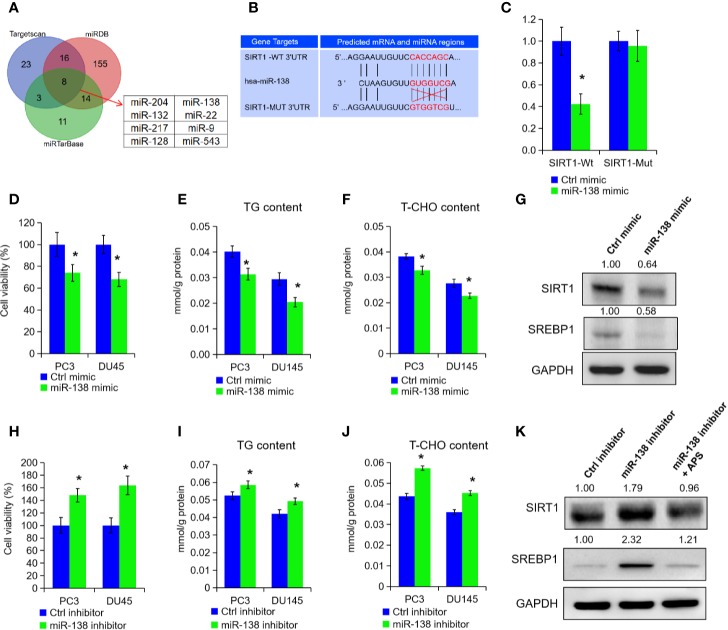
miR-138-5p can specifically bind to SIRT1 to promote tumor progression. The possible miRNAs of SIRT1 were analyzed in online database (i.e. Targetscan, miRTarBase, and miRDB). The Venn analysis showed that eight miRNAs were included in above three data sets **(A)**. The miR-138-5p binding site located on the 3′UTR of SIRT1 mRNA in wild type (SIRT1-Wt) or mutant (SIRT1-Mut) were cloned into the pGL3-basic-Vector **(B)**. The luciferase activities in cells transfected with the SIRT1-Wt and SIRT1-Mut plasmids and miR-138-5p were measured on the multifunctional microplate reader **(C)**. Cell viability, TG, and T-CHO were assessed in PC3 and DU145 cells transfected with miR-138-5p mimic or inhibitor **(D–F, H–J)**. The protein levels of SIRT1 and SREBP1 were detected in PC3 cells transfected with miR-138-5p mimic or inhibitor **(G**, **K)**. Asterisk (*) indicates P < 0.05.

To explore the post-transcriptional modification of SIRT1 in PCa, we searched three online bioinformatics websites to predict potential miRNA targets of SIRT1, i.e. Targetscan, miRDB, and miRTarBase. After the Venn analysis of those miRNA target, we found a total of eight miRNAs in above three data sets, and miR-138-5p was identified finally for further experiments (**Figures 4A, B**). Subsequently, we constructed two SIRT1 luciferase reporter plasmids, and the results confirmed that miR-138-5p could specifically bind the 3′UTR region of SIRT1 and inhibit the expression of SIRT1 (**Figures 4B, C**, P < 0.05). When SIRT1 was mutated at 3′UTR region specific sites, miR-138-5p could not bind to SIRT1 and could not inhibit the expression of SIRT1 (**Figures 4B, C**). Furthermore, we transfected miRNA-138-5p mimic into PC3 and DU145 cells to overexpress miR-138-5p levels. As shown in **Figures 4D–F**, the cell growth, TG, and cholesterol level were decreased upon miR-138-5p overexpression (P < 0.05). Also, Western blot assay expounded that the protein levels of SIRT1 and SREBP1 expression in PC3 cells were reduced under miR-138-5p overexpression (**Figure 4G**). On the contrary, we inhibited the miR-138-5p levels in PC3 and DU145 cells after transfection of miR-138-5p inhibitor. Cell proliferation ability, TG, and cholesterol level were significantly increased in PC3 and DU145 cells after the suppression of miR-138-5p (**Figures 4H–J**, P < 0.05). The protein levels of SIRT1 and SREBP1 were greatly increased under miR-138-5p knockdown (**Figure 4K**). Additionally, APS treatment restrained the elevated expression of SIRT1 and SREBP1 under miR-138-5p knockdown (**Figure 4K**). In summary, the above data revealed that SIRT1 might be involved in tumor process as a downstream target gene of miR-138-5p under APS treatment.

## Discussion

Epidemiological studies suggested that a high-fat diet plays a critical role in the development of PCa (Ferro et al., 2017; Narita et al., 2019). Further findings indicated deregulated lipid metabolism was positively associated with advanced PCa. Until now, some agents targeting multiple metabolic pathways or AR pathways have been used into pre-clinical studies to improve “cancer kill” and reduce the toxic side effect (Butler et al., 2016; Galbraith et al., 2018).

Because of the disadvantages of statins in reducing serum levels, Traditional Chinese Medicine have been applied increasingly for their superiority in regulating lipid metabolism (Li et al., 2017). In the present study, we found APS, extracted from *Astragalus*, significantly decreased the PCa cell growth, cellular TG, and cholesterol levels in a dose-dependent and time-dependent manner. Besides, tumor-bearing mice experiments also confirmed that a significant suppression of tumor growth, TG, and total cholesterol (T-CHO) levels in mice after oral administration of APS. Unfortunately, no significant difference between HD-APS and LD-APS groups in serum TG and T-CHO levels were found in above experiment. Besides, we also performed the Nile red and Oil red staining to detect the lipid droplet in above tumor-bearing model. Unfortunately, we did not find any difference or variation trend in the neither HD-APS nor LD-APS group. As a result, we wonder whether that higher concentration of APS consumption may cause significant changes in fatty acid staining. Then, we performed two additional concentration of APS (i.e. 200 and 400 mg/kg) in our *in vivo* experiments, and the results showed several mice appear to die in above groups. Thus, we thought there might be additional anti-tumor signaling (other than lipid metabolism) in the above process. All in all, Chinese herbal medicine has drawn a great attention in regulating lipid metabolism and prevention of caner progression.

Our gene expression profiling results indicated that APS treatment altered the expression of genes involved in inflammation, cell cycle, and metabolism. Furthermore, we identified that APS enhances expression of SIRT1, which is important for lipid metabolism. Type III histone deacetylase SIRT1 is NAD+ dependent enzyme, participating in cell genome stability maintenance, lipid metabolism, and various biological processes (Yang et al., 2015; Ye et al., 2017). In addition, SIRT1 also take part in regulating the expression of genes involved in tumorigenesis by deacetylating both histone and non-histone targets (Alves-Fernandes and Jasiulionis, 2019). In PCa, elevated expression of SIRT1 has also been reported to promote cancer cells growth, invasion and neuroendocrine differentiation (Cui et al., 2016; Ruan et al., 2018). Besides, overexpression of Sirt1 in mesenchymal stem cells inhibits PCa cells growth prostate growth through recruiting natural killer cells and macrophages in tumor micro-environment (Yu et al., 2016). Lactate uptake stimulates SIRT1-dependent PGC-1α activation and mitochondrial mass and activity in PCa cells and cancer-associated fibroblasts (Ippolito et al., 2019). In our study, APS treatment inhibits SIRT1 mRNA and protein levels in PCa cells. Further molecular investigation showed that SIRT1 promotes the expression and nuclear translocation of SREBP1 *via* activating AMPK phosphorylation and then inhibits cellular lipid metabolism.

Various studies indicated that some miRNAs related with SIRT1, such as miR-204, miE-212/132, miR-449a, miR-221/222, and miR-34a, induce cell growth, invasion, and angiogensis in PCa (Karbasforooshan et al., 2018; Kasomva et al., 2018). Through network prediction, we verified that histone deacetylase SIRT1 3′UTR region has miR-138 specific binding sites, and the high expression of miR-138 can significantly inhibit SIRT1 mRNA and protein expression levels of PCa cells. In addition, we found that APS treatment increased the levels of miR-138-5p to inhibit cancer cell growth, TG, and cholesterol levels *in vitro*. As a tumor suppressor, miR-138 can inhibit oncogenic proteins by directly bind to their mRNAs in multiple caner types, including glioblastoma, non-small cell lung cancer, and PCa (Yeh et al., 2019). In 2013, Gong et al. noted that inhibition of miR-138 induced lipid raft formation by constitutive NF-κB activation and upregulating many components of lipid rafts (Gong et al., 2013). Even though SIRT1 has been found as a potential target of miRNA-138 in macrophages, smooth muscle cells, endothelial cells, and tumor cells, no data has been published in PCa concerning miR-138 and SIRT1 expression (Xu et al., 2015; Zhu et al., 2017; Bai et al., 2018). In current study, our results showed that miR-138-5p could specifically bind the 3′UTR region of SIRT1 and inhibit the expression of SIRT1 in PCa cells.

All together, we found APS treatment greatly inhibited cell growth, triglyceride, and cholesterol levels of PCa cells *in vitro* and *in vivo*. Mechanistic investigation demonstrated that decreased expression of SIRT1 inhibits the expression and stabilization of SREBP1 to suppress cellular lipid metabolism. In addition, we also found miR-138-5p was greatly inhibited the SIRT1 expression by targeting its 3′UTR region. To summarize, our findings suggested that APS inhibits tumorigenesis and lipid metabolism through miR-138-5p/SIRT1/SREBP1 pathways in PCa.

## Data Availability Statement

The raw data supporting the conclusions of this article will be made available by the authors, without undue reservation, to any qualified researcher.

## Ethics Statement

The animal study was reviewed and approved by the Ethics Committee of First Teaching Hospital of Tianjin University of Traditional Chinese Medicine.

## Author Contributions

YJ and XL designed the experiment. SG and BM wrote the manuscript and performed experiments. XJ contributed to the reagents and materials. XJ revised the manuscript.

## Funding

This work was supported by grants from the National Natural Science Foundation of China (No. 81603438, 81802568)

## Conflict of Interest

The authors declare that the research was conducted in the absence of any commercial or financial relationships that could be construed as a potential conflict of interest.
