# Supplementary material for: Astragalus Polysaccharides Inhibits Tumorigenesis and Lipid Metabolism Through miR-138-5p/SIRT1/SREBP1 Pathway in Prostate Cancer
Source: Front Pharmacol. 2020 May 5;11:598. doi: 10.3389/fphar.2020.00598 (PMC7214922; doi:10.3389/fphar.2020.00598)
Supplement: Supplementary file 1 [file DataSheet_1.doc]

Supplemental Table 1 **The sequences of qRT-PCR and shRNA**

| Gene Name | Sequences(5'-3') of qRT-PCR |
| --- | --- |
| SIRT1 F | TGTGTCATAGGTTAGGTGGTGA |
| SIRT1 R | AGCCAATTCTTTTTGTGTTCGTG |
| SREBP1 F | ACAGTGACTTCCCTGGCCTAT |
| SRBEP1 R | GCATGGACGGGTACATCTTCAA |
| ACC F | ATGTCTGGCTTGCACCTAGTA |
| ACC R | CCCCAAAGCGAGTAACAAATTCT |
| FASN F | AAGGACCTGTCTAGGTTTGATGC |
| FASN R | TGGCTTCATAGGTGACTTCCA |
| GAPDH F | TGACCCCTTCATTGACCTCA |
| GAPDH R | AGTCCTTCCACGATACCAAA |
|  |  |
| Gene Name | Sequences(5'-3') of shRNA |
| Sirt1-F | CCGGCAGGTCAAGGGATGGTATTTACTCGAGTAAATACCATCCCTTGACCTGTTTTTG |
| Sirt1-R | AATTCAAAAACAGGTCAAGGGATGGTATTTACTCGAGTAAATACCATCCCTTGACCTG |

Supplemental Table 2 **The gene list of microarray analysis (Fold change ≥±3)**

| No. | Gene Name | Fold change |  | No. | Gene Name | Fold change |
| --- | --- | --- | --- | --- | --- | --- |
| 1 | C11orf97 | 7.37 |  | 1 | CXCL13 | -5.47 |
| 2 | TLR1 | 6.24 |  | 2 | LEKR1 | -3.92 |
| 3 | OR7A5 | 5.78 |  | 3 | CCL16 | -3.82 |
| 4 | PDGFRB | 4.69 |  | 4 | LOX | -3.71 |
| 5 | SCN7A | 4.47 |  | 5 | ADAMTS12 | -3.68 |
| 6 | LINC01069 | 4.22 |  | 6 | NBPF25P | -3.55 |
| 7 | MASP1 | 4.17 |  | 7 | CCM2L | -3.31 |
| 8 | SH3BP5L | 3.93 |  | 8 | NAALADL2 | -3.12 |
| 9 | ZSCAN31 | 3.63 |  |  |  |  |
| 10 | FUT2 | 3.58 |  |  |  |  |
| 11 | SIRT1 | 3.46 |  |  |  |  |
| 12 | LPPR4 | 3.35 |  |  |  |  |
| 13 | CACNG3 | 3.28 |  |  |  |  |
| 14 | LOC101928769 | 3.23 |  |  |  |  |
| 15 | COL8A1 | 3.17 |  |  |  |  |
| 16 | OR4C16 | 3.14 |  |  |  |  |
| 17 | CAV1 | 3.12 |  |  |  |  |
| 18 | RAG2 | 3.10 |  |  |  |  |
| 19 | PIEZO2 | 3.04 |  |  |  |  |
| 20 | KANK4 | 3.01 |  |  |  |  |
